# Perceptions of students regarding E-learning during Covid-19 at a private medical college

**DOI:** 10.12669/pjms.36.COVID19-S4.2766

**Published:** 2020-05

**Authors:** Sahar Abbasi, Tahera Ayoob, Abdul Malik, Shabnam Iqbal Memon

**Affiliations:** 1 Dr. Sahar Abbasi, MCPS-HPE. Department of Medical Education, College of Physicians and Surgeons Pakistan, Karachi, Pakistan; 2 Dr. Tahera Ayoob, FCPS (OMFS). Department of Oral & Maxillofacial Surgery, Liaquat College of Medicine and Dentistry, Karachi, Pakistan; 3 Dr. Abdul Malik, , MD & DCN (Neurology). Department of Medicine, Liaquat College of Medicine and Dentistry, Karachi, Pakistan; 4 Dr. Shabnam Iqbal Memon, Department of Medical Education, College of Physicians and Surgeons Pakistan, Karachi, Pakistan

**Keywords:** Covid-19, E-learning, Face-to-face teaching, Perceptions

## Abstract

**Objective:**

The purpose of this study was to determine the perceptions of students towards e-learning during the lock down.

**Methods:**

A descriptive cross-sectional study was conducted at Liaquat College of Medicine and Dentistry. MBBS and BDS students of all levels participated in the study with a sample size calculated as 377. A self-administered questionnaire was developed. After validation from the Medical Education Experts, pilot test was run on 30 participants before the administration of the questionnaire. The questionnaire was emailed to the participants for data collection. Reliability of the questionnaire was determined. Independent T-test was applied for determining the perceptions of students towards e-learning. Frequencies and percentages were also computed for demographics.

**Results:**

Total 382 responses were received.137 males and 245 females participated in the study. 0.851 was calculated as Cronbach’s alpha of the questionnaire. Overall, 77% students have negative perceptions towards e-learning. 76% of the students use mobile device for their e-learning.

**Conclusion:**

Students did not prefer e-teaching over face-to-face teaching during the lock down situation. Administration and faculty members should take necessary measures for improving e-teaching for better learning during lock down.

## INTRODUCTION

The magnanimity of information technology’s influence on multiple aspects of our lives today cannot be refuted, neither can its growing popularity and use in the education sector be denied.^1^ This role in the academic arena has gained importance furthermore considering the ongoing Covid-19 pandemic causing all educational institutions the world over to close down and thus giving rise to multiple challenges at all stages and levels of education in particular for students.^2^ The flourishing innovative technologies and learning management systems both for teaching and assessment have taken a headway providing a utilizable solution for educators and giving policy makers an opportunity to implement the use of information technology during the quarantine days for covering the course work.^3^ Stakeholders involved including institutional administrators, teachers, students, etc. are making considerable efforts to optimally utilize the available technology for continuing the process of education and minimizing the gaps that are going to result as a consequence of the current circumstances.^1^,^4^

There are several studies based on the significance and efficacy of implementation of e-learning.^1^,^3^,^5^ Many universities across the world are promoting it as a teaching method and it is being widely appreciated by the learners.^5^,^6^ There are numerous reasons for its overall acceptability; few of them particularly applicable in case of learners are its ease of use, flexibility and better control over the environment. However, in spite of its multiple advantages there are quite a few limitations of e-learning such as social isolation, lack of student-teacher interaction and connectivity issues etc.^7^-^9^

Despite the wide-based adoption of e-learning the world over, it was never considered as a part of formal education in Pakistan by majority of the institutions until the spread of Covid-19 recently.^9^,^10^ Due to the lockdown situation however, now a lot of schools, colleges and even undergraduate medical and dental institutes across the country are moving towards e-learning. Medical and Dental Colleges’ administrators and teachers are taking appropriate measures to conduct effective e-learning via e-lectures, e-tutorials, e-case based learning, etc. so that continued education can be provided without getting much affected during the quarantine period.^7^,^8^,^11^ Also various e-teaching softwares are being explored by teachers to bring maximum possible ease for their students.

Considering the relatively recent advent of this teaching methodology in Pakistan, both teachers and students are still in the process of getting acquainted with the new system.^9^ At this point in time, it is important to find out students’ opinion and viewpoint regarding this virtual approach to teaching and learning. Whether the learners are attuned to the new methodology, would prefer any modifications, or rather would want to go back to conventional learning altogether, would be an interesting point to explore.^7^,^12^ Therefore, the purpose of this study was to determine the perceptions of students towards e-learning during the lock down due to covid-19.

## METHODS

After taking approval from the Ethical Review Committee of the College (Ref.No.EC/33/20, dated April 18, 2020), a cross-sectional descriptive study was conducted in April 2020 on the students of Liaquat College of Medicine and Dentistry. Total strength of students in the College was 800 consisting of 300 BDS and 500 MBBS students. Raosoft software was used for calculating the sample size.^13^ Keeping the margin of error at 3.68%, confidence interval at 95% and population size of 800, the sample size was calculated as 377. Convenience sampling technique was used to select the participants for the study. A self-administered questionnaire was developed through literature search. It had 23 items all together. The scale was based on 5-point likert scale: 1- strongly disagree, 2- disagree, 3- Somewhat agree 4-agree, 5- strongly agree. 5 items of the questionnaire covered demographics, one item was to determine the choice of gadgets used for e-learning, 17 items of the questionnaire determined the positive and negative perceptions of students towards e-learning. Ten of the 17 items were negatively worded and were reversely scored. Before administration of the questionnaire, validation by two medical educationists was done. Pilot test was also run on 30 participants. Reliability of the questionnaire was calculated and turned out to be 0.85.

For further understanding of the data, 17 items of the questionnaire were grouped into 5 categories such as Future learning preference, E-teaching is better than traditional teaching, Quality of e-teaching is satisfactory, Impact of e-learning is less, Student-Teacher interaction (isolation has increased), Online teaching is not secure. The questionnaire was emailed to all the students for the collection of data. Written informed consent was also taken from the participants.

SPSS version 23 was used for data analysis. A Mean was calculated for 17 items with scores ranging from 17-85. The Mean score came out to be 43. Those who scored less than the Mean (less than 43) were considered having positive attitude and those with score of more than 43 were considered to have negative attitude towards e-learning. 17 items were divided into 5 groups with following mean values where less than mean would depict positive and more than mean negative attitude:

- “Future learning preference” - 5 items (Score min 5 & max 25, Mean 13),
- “E-teaching is better than traditional teaching” -4 items (Score min 4 & max 20, Mean 10),
- “Quality of e-teaching is satisfactory”-2 items (Score min 2 & max 10, Mean 5),
- “Impact of e-learning is less”-1 item (Score min 1 & max 5, Mean 3)
- “Student-Teacher interaction (isolation has increased) - 1 item (Score min 1 & max 5, Mean 3),
- “Online teaching is not secured-1 item (Score min 1 & max 5, Mean 3).

Independent T-test was applied for data analysis. Frequencies and percentages were computed for the demographics. Cronbach’s Alpha (reliability) was also determined for all items of the questionnaire.

## RESULTS

A total of 382 MBBS and BDS students participated in the study. The demographics of the participants along with the choice of gadgets used for their e-learning are shown in Table-I. It was found that 76% of the students use Mobile for their e-learning. 75.7% of the students have negative perceptions towards e-learning

**Table-I:** Demographics and choice of gadgets used for e-Learning.

	Frequency	Percentage %
***Gender***		
Male	137	35.9%
Female	245	64.1%
***Discipline***		
MBBS	204	53.4%
BDS	178	46.6%
***Year***		
First year	97	25.4%
Second Year	87	22.8%
Third Year	81	21.2%
Fourth Year	88	23%
Fifth Year	29	7.6%
***Choice of Gadget/Device***		
Mobile	289	75.7%
Computer	3	0.8%
Laptop	81	21.2%
Tablet	9	2.4%

The overall perception and category wise responses of students towards e-learning along with their respective p-values are shown in Table-II. As computed in the pilot study of 30 responses the reliability score of the questionnaire was 0.851.

**Table-II:** Overall perception and category wise responses of students towards E-learning N=382.

	Responses	N(%)	Mean	Std. Deviation	Std. Error Mean	p-value
***Overall Perception***	Positive	86 (23%)	37.5455	6.27540	1.09241	0.015
	Negative	296 (77%)	58.7249	8.16173	0.43689	
***Future learning Preference***	Positive	86 (23%)	4.2791	0.86298	0.09306	0.000
	Negative	296 (77%)	7.9392	1.38626	0.08057	
***E-teaching is better than traditional teaching***	Positive	56 (15%)	8.5000	1.82906	0.24442	0.000
	Negative	326 (85%)	14.7546	2.56387	0.14200	
***Quality of e-teaching is satisfactory***	Positive	119 (31%)	7.6134	0.66523	0.06098	0.000
	Negative	263 (69%)	10.2586	1.28470	0.07922	
***Impact of e-learning is less***	Positive	327 (86%)	18.2508	2.87118	0.115878	0.000
	Negative	55 (14%)	11.4545	1.87398	0.25269	
***Student-Teacher interaction isolation has increased***	Positive	320 (84%)	2.2875	0.63727	0.03562	0.000
	Negative	62 (16%)	4.1935	0.39830	0.05058	
***Online teaching is Not secured***	Positive	241 (63%)	1.9149	0.28003	0.02358	0.000
	Negative	141 (37%)	3.9087	0.77994	0.05024	

## DISCUSSION

Our study indicates that out of 382 students, 76% of them used mobile gadgets for their e-learning. 77.4% students showed negative perception about e-learning, out of which 86% students felt e-learning has little impact on their learning. Majority of the students preferred face to face teaching over e-teaching. The key outcome of the result shows that the students are not yet ready for e-learning.

Mobile has become one of the most popular devices among students for e-learning as compared to laptops and tablets.^14^ In one of the studies conducted on university students,^15^ it was found that 66% use mobile devices for e-learning, which is very similar to our study that shows 76% students prefer mobile devices. A research conducted in Spain revealed that students chose mobile for their learning because student-teacher interaction through mobile was much easier as compared to other devices.^16^ Another very common reason for this is that learning can take place anytime and anywhere as discussed in the article by Angela Murphy and her co-authors.^17^ The results of this study were slightly different from ours as mobile was the second choice for e-learning after laptop, whereas, in our case laptop’s preference came at number two after mobile.

Post Covid-19 outbreak, students in Pakistan were required to move to online learning, however, they have found it less appealing due to its limitations with respect to practical aspects of learning in the lab/clinical environment. This is consistent with the students’ behavior in many other countries like China, Malaysia, Singapore etc.^18^-^20^

When other literature was searched regarding e-learning under normal circumstances, before Covid-19, we came across mixed outcomes. Some suggested positive and others negative inclination towards e-learning.

Singh A, Min AK did a study on the efficacy of conducting digital lectures on gross anatomy. The study investigated student’s satisfaction level towards e-learning and it was found that majority of the students accepted digital learning.^21^ Raymond Selorm also revealed in his paper that in comparison to face to face learning students were satisfied with e-learning.^22^ However, there also exists literature that reports students preferring face to face teaching over online teaching^23^,^24^ like in our study wherein students preference was 85%.

Our study results also highlighted that students are not ready to adopt e-learning which is different than the findings of a study conducted on nurses. They considered e-learning as a better teaching tool and preferred it for future learning.^25^ Another study conducted in India by Sunita and Colleagues revealed that e-teaching increased students’ satisfaction level towards learning.^8^

One of the studies also pointed out that students were misusing the user identity during online teaching. It was noted that online teaching was not secure as incivility was considered as a major issue that was detrimental to students’ privacy, as is evident in our findings also.^26^

There are several studies on the comparison of e-learning with face to face teaching.^27^,^28^ In one of the papers presented in a conference on mobile learning at Singapore, it was reported that there is no significant difference between the performance of students taught by e-learning and face to face learning, whereas in our study it was found that e-learning is perceived to have little impact compared to face to face learning as indicated by 86% of the participants. The same paper that was presented in Singapore also highlighted that e-teaching methodology limits student-teacher interaction.^27^ This finding was in congruence with our study where 84% of the students rated that e-teaching has limited student-teacher interaction.

### Limitations of the study

One of the limitation of the study is that sample population has been drawn from a single, private medical and dental college. Therefore, results of the study cannot be generalized.

## CONCLUSION

It is concluded that in Pakistan, despite gaining immense popularity today, digital technology has still not been embraced by the Medical and Dental students for use in teaching. Students are still more inclined towards face to face teaching rather than e-teaching. Administration and faculty members should take necessary measures for improving e-teaching quality to help with better learning of students during lock down.

### Recommendation

The recommendation of the study is to further explore factors influencing students perceptions towards e-learning. It is also recommended to explore the perceptions of Faculties regarding their experience towards e-teaching during covid-19 lockdown.

### Authors’ Contribution

**SA** concert, design, data collection and data analysis, drafting, editing of manuscript, final review, integrity of research.

**TA** data collection and data analysis, drafting, statistical analysis.

**AM** data collection, final review.

**SIM** manuscript review and editing.
